# SMART MUSIC INTERVENTION: PROTOCOL DEVELOPMENT OF A WEARABLE DEVICE FOR COLLECTION OF PHYSIOLOGICAL DATA

**DOI:** 10.1093/geroni/igad104.2867

**Published:** 2023-12-21

**Authors:** Emily Ihara, Parth Pathak, Megumi Inoue, Catherine Tompkins, Y Alicia Hong

**Affiliations:** George Mason University, Fairfax, Virginia, United States; George Mason University, Fairfax, Virginia, United States; George Mason University, Fairfax, Virginia, United States; George Mason University, Fairfax, Virginia, United States; George Mason University, Fairfax, Virginia, United States

## Abstract

This study tested a protocol of collecting physiological data through sensors on a wearable device in response to favorite music from late adolescence/early adulthood. The benefits of personalized music for individuals living with dementia have been demonstrated in various studies, showing decreases in agitation, anxiety, depression, and other behavioral and psychological symptoms of dementia and improvement in mood, memory, and well-being. Most studies have relied on observational data, standardized clinical scales, and self- or caregiver-reported perceptions of the effects of personalized music. Passively-collected physiological data may minimize researcher bias during data interpretation. We tested the protocol on 15 participants, triangulating the physiological, observational, and self-reported effects of personalized music on stress. Physiological measures were captured by a wearable wristband Empatica E4 and included heart rate, electrodermal activity, and temperature. Self-reported measures included the Perceived Stress Scale, level of stress on a 0-10 scale, and reactions to each song (happy, energized, calm, relaxed, nostalgic, sad, excited). Prior to music listening, we induced stress through three iterations of the Trier Mental Challenge Test, which involves three minutes of math exercises. Participants reported their level of stress after the Trier Test and after each song. Researchers observed participant reactions during each song. Preliminary analysis shows that nine minutes of a stress-inducing activity was enough to capture stress reactions and demonstrated a feasible protocol for collecting physiological, observational, and self-report data. These results inform the development of a novel protocol that responds to a person’s stress levels through a personalized music listening intervention.

